# tRF-1-ArgTCG-1-1 promotes renal fibrosis by regulating β-catenin

**DOI:** 10.1080/0886022X.2026.2670055

**Published:** 2026-06-01

**Authors:** Ling Jin, Ci Wang, Yuye Yan, Yuye Chen, Zihao Xiao, Yajie Wang, Yuanhui Shi, Cong Fu, Yuhan Cao

**Affiliations:** ^a^ Department of Nephrology, Yi Ji Shan Hospital Affiliated to Wannan Medical College, Wuhu, China; ^b^ Anesthesia Laboratory and Training Center of Wannan Medical College, Wuhu, China; ^c^ Anhui Province Key Laboratory of Non-coding RNA Basic and Clinical Transformation, Wuhu, China; ^d^ Department of Cardiology, Yi Ji Shan Hospital Affiliated to Wannan Medical College, Wuhu, China

**Keywords:** Chronic kidney disease, tRFs, mechanism, renal fibrosis, β-catenin

## Abstract

**Background:**

Renal fibrosis (RF) is a crucial pathological factor in the progression of chronic kidney disease (CKD) to end‐stage renal failure. The role of noncoding RNAs (ncRNAs) in CKD has been researched recently. tRNA-derived fragments (tRFs) constitute a class of small ncRNAs originating from tRNAs. To date, the function of tRFs in RF remains unexplored.

**Methods:**

Human small RNA microarray analysis was performed to identify dysregulated tRFs in urine-derived renal tubular epithelial cells from patients with RF. RT-PCR was used for validation in clinical samples. Hydroxylamine-mediated protein precipitation mass spectrometry (Hypro-MS), parallel reaction monitoring (PRM), and antisense oligonucleotide (ASO)-mediated knockdown were used to investigate the interaction between tRF-1-ArgTCG-1-1 and β-catenin and its functional role in TGF-β1-treated HK-2 cells.

**Results:**

Microarray profiling identified tRF-1-ArgTCG-1-1 as an upregulated tRF in urine-derived renal tubular epithelial cells from patients with RF. RT-PCR further confirmed that tRF-1-ArgTCG-1-1 expression was significantly increased in urine samples from patients with CKD and was associated with impaired renal function and a higher risk of RF. Hypro-MS and PRM analyses demonstrated an interaction between tRF-1-ArgTCG-1-1 and β-catenin. ASO-mediated silencing of tRF-1-ArgTCG-1-1 reduced β-catenin expression in HK-2 cells. Moreover, silencing of tRF-1-ArgTCG-1-1, alone or together with β-catenin, attenuated TGF-β1-induced expression of RF-related markers *in vitro*.

**Conclusions:**

Overall, these findings suggest that renal tubular epithelial cells tRF-1-ArgTCG-1-1 play a key role in RF and that the tRF-1-ArgTCG-1-1/β-catenin pathway is a potential therapeutic target for CKD and RF.

## 1. Introduction

Chronic kidney disease (CKD) is a major public health problem, with a global prevalence of 13–15% [1–3]. Renal fibrosis (RF) is a common pathological feature of CKD and ultimately leads to renal dysfunction through tubular degeneration, inflammation, interstitial fibroblast activation, and extracellular matrix protein deposition [4,5]. Although there is a certain understanding of the mechanism of RF, there remains a lack of effective treatments for CKD, making finding therapeutic approaches that can alleviate the progression of CKD crucial.

Over the years, many studies have focused on elucidating the roles of noncoding RNAs (ncRNAs). Compared with other classes of ncRNA, tRNA has been largely overlooked in functional studies. tRFs were initially considered random byproducts of tRNA degradation [6], but increasing evidence has demonstrated that they play significant biological roles, suggesting previously unrecognized regulatory functions.

Recent studies have identified tRNA-derived small noncoding RNAs (tsRNAs), including tRNA halves (tiRNAs) and tRNA-derived fragments (tRFs), which have now become a focus of disease research [7]. Notably, tRFs are involved in transcriptional regulation and translational repression [8,9]. Studies have demonstrated that tRFs are indispensable for cellular function [10,11]. Specifically, 5′-tRNAAla exerts a neuroprotective effect through the G-quadruplex structure [12]. Abnormal expression of tRFs has been observed in many diseases, including tumors, neurodegenerative diseases and metabolic and infectious diseases [13,14].

Importantly, recent studies have further highlighted the emerging roles of renal tRFs/tsRNAs in kidney diseases. Newly published evidence suggests that tRFs/tsRNAs participate in high glucose-induced podocyte injury, diabetic kidney disease progression, extracellular matrix regulation, and renal stress-protective pathways [15,16]. In addition, recent review articles have summarized the biological functions and potential clinical relevance of tsRNAs in kidney diseases [17]. These findings indicate that renal tRF/tsRNA research is rapidly evolving and underscore the importance of further investigating their roles in CKD and RF.

Recent work has revealed that tRFs are also involved in kidney diseases [18,19]. Yin et al. reported that tRF-Val-TAC-004 prevented renal ischemia–reperfusion injury by attenuating Apaf1-mediated apoptosis in BUMPT cells [20,21]. However, the role of tRFs in RF has not been systematically elucidated. In this study, we explored the correlation between renal tubular epithelial cell-derived tRFs and RF and elucidated the mechanism by which tRFs are involved in RF.

## 2. Materials and methods

### 2.1. Study population

This study was conducted from January 2023 to January 2025 at the Department of Nephrology, Yi Ji Shan Hospital, Wannan Medical College. The morning urine samples (approximately 100 mL) from three patients with CKD without RF and three patients with CKD with RF were collected in this study. Second, whole RNA from urine-derived renal tubular epithelial cells was isolated for analysis via a human tRF microarray. Then, morning urine samples from a total of 66 patients with CKD of different stages and pathological types who underwent renal puncture biopsy were collected. Whole RNA from urinary renal tubular epithelial cells was isolated for analysis via real‐time quantitative PCR (RT-qPCR). All 66 patients with CKD were divided into a nonfibrosis group, a mild to moderate fibrosis group and a severe fibrosis group according to the tubulointerstitial fibrosis score. The selection criteria for patients with CKD were as follows: patients with abnormal renal structure or function for more than 3 months according to the 2024 KDIGO guidelines; patients with stages G1–G5 CKD; patients whose glomerular filtration rate (GFR) had decreased to less than 60 mL/min/1.73 m^2^ within the past 3 months; patients with persistently elevated urinary protein/albumin levels (such as the albumin–creatinine ratio (ACR) in the urine ≥30 mg/g); and patients with relatively complete clinical data and good compliance with medical advice. The exclusion criteria for patients with CKD were as follows: patients who had recently experienced major surgery or other serious illnesses (such as acute urinary tract infections and cancer); patients with illnesses that could impact research outcomes, such as severe cardiac insufficiency, severe hepatitis, and psychological problems; patients on drugs that could affect study findings, such as immunosuppressants and strong antihypertensive drugs; patients who exhibited postrenal transplant rejection responses or allergy reactions while in the hospital; and patients in particular groups, such as lactating women and pregnant women.

### 2.2. Collection of clinical and pathological data

We collected basic demographic and clinical biochemical data, such as age, sex, blood pressure, serum creatinine (Scr) level, cystatin C level, estimated glomerular filtration rate (eGFR), and 24-h urine protein, from patients with CKD. Moreover, renal histopathological data were acquired. Paraffin-embedded renal tissues were used to prepare 4 μm sections for Masson staining. Subsequently, 20 nonoverlapping fields per section were randomly chosen under ×200 magnification and imaged. Renal fibrosis was scored as follows: fibrosis that affected ≤25% of the area (no fibrosis); fibrosis greater than 26% and fibrosis less than 50% (mild to moderate fibrosis); fibrosis that affected >50% of the area (severe fibrosis).

### 2.3. Isolation of renal tubular epithelial cells by immunomagnetic beads

Morning urinary samples (100 mL) were obtained from the included patients with CKD. CD13^+^ renal tubular epithelial cells were generated according to the methods reported in our previous study [22]. The expression of AQP-1 was measured with Western blotting to identify renal tubular epithelial cells.

### 2.4. Human small RNA array analysis

Urine samples from three patients with RF and urinary samples from three patients without RF were selected. After CD13^+^ cells were separated from the urinary sediment, total RNA was extracted using TRIzol‐LS reagent (Thermo Fisher Scientific, Waltham, MA) following the manufacturer’s instructions. Arraystar small RNA microarray analysis was performed by Aksomics Biotechnologies (Shanghai, China). Differentially expressed small RNAs between the two comparison groups were identified by fold change (FC) and statistically significant (*p* value) thresholds. Hierarchical clustering heatmaps, scatter plots, and volcano plots were generated to display small RNA expression patterns among samples by R software (R Foundation for Statistical Computing, Vienna, Austria).

### 2.5. Cell culture, antisense oligonucleotide (ASO), and siRNA transfection

HK-2 cells were obtained from Procell (Pricella, Wuhan, China). The cells were cultured in complete HK-2 cell-specific medium (Dulbecco’s modified Eagle’s medium + 10% fetal bovine serum + 1% 100 U/mL penicillin and 100 μg/mL streptomycin; Pricella, Wuhan, China, BL304A). The cells were maintained in a humidified atmosphere at 37 °C with 5% CO_2_.

The cells were seeded in cell culture plates. When the cells reached 40–60% confluency, they were transfected with 20 nM tRF-1-ArgTCG-1-1 ASO (GenePharma, Shanghai, China) or siRNA-β-catenin (GenePharma, Shanghai, China) with Lipofectamine 2000 transfection reagent (SignaGen, Frederick, MD). Then, the cells were cultured for 24 h. HK-2 cells (1 × 10^7^) were then exposed to 15 ng/mL recombinant TGF-β1 protein (Sino Biological, Beijing, China, 50698-M08H-B) for 48 h. Total RNA or protein was collected from the cells.

### 2.6. Immunofluorescence staining

Cell‐containing small dishes were fixed with 4% paraformaldehyde and permeabilized with 0.3–0.5% Triton X‐100. Collagen I (diluted 1:100; Abcam, Shanghai, China, AB270993), β-catenin (diluted 1:500; Proteintech, Rosemont, IL, 66379-1-IG), and α‐SMA (diluted 1:100; Proteintech, Rosemont, IL, 14395-AP) primary antibodies were used for staining. The secondary antibodies used were Alexa Fluor 647‐conjugated anti‐rabbit IgG (Invitrogen, Carlsbad, CA, ab190565) and Alexa Fluor 647‐conjugated anti‐mouse IgG (Invitrogen, Carlsbad, CA). 4,6-Diamidino-2-phenylindole (DAPI, Thermo Fisher, Waltham, MA, C1002) was used to stain the nuclei. Confocal microscopy (Leica, Wetzlar, Germany) was used to obtain images.

### 2.7. RNA pretreatment, cDNA synthesis, and RT-qPCR for tRF-1-ArgTCG-1-1

Total RNA was pretreated with the rtStar™ tRF&tiRNA Pretreatment Kit (Cat# AS-FS-005, Arraystar, Rockville, MD) to remove the modifications and then reverse transcribed into cDNA via the rtStar™ First‐Strand cDNA Synthesis Kit (Cat# AS-FS-003-02, Arraystar, Rockville, MD) according to the manufacturer’s protocol. qRT-PCR was performed using 2X PCR master mix (Arraystar, Rockville, MD). The reactions were performed with a real-time PCR system. The average expression levels of urinary tRFs were normalized against U6, and the levels of tRFs were calculated via the 2^−ΔΔCt^ method for relative quantification of expression, in which ΔCt = Ct(tRFs) − Ct(U6) and ΔΔCt = ΔCt(case) − ΔCt(control). The primary primers used were as follows:

U6: forward 5′-GCTTCGGCAGCACATATACTAAAAT-3′, reverse 5′-CGCTTCACGAATTTGCGTGTCAT-3′
tRF-1-ArgTCG-11-1: forward 5′-CGACGATCAAGGGAGGTTATG-3′, reverse 5′-CGTGTGCTCTTCCGATCTAAAGT-3′

### 2.8. Western blot

Protein was extracted from cells using phenylmethanesulfonyl fluoride (PMSF) and radioimmunoprecipitation assay (RIPA) lysis buffers (Beyotime, Shanghai, China). Protein concentrations were measured with a bicinchoninic acid (BCA) kit (Beyotime, Shanghai, China, P0009). Whole extracts were separated by 10% sodium dodecyl sulfate–polyacrylamide gel electrophoresis (SDS–PAGE). The protein samples were subsequently subjected to electrophoresis and transferred to a PVDF membrane. After blocking with 5% bovine serum albumin, primary antibodies, including rabbit collagen I (diluted 1:1,000; Abcam, Shanghai, China, AB270993), α-SMA (diluted 1:3,000, Proteintech, Rosemont, IL, 14395-1-AP), β-catenin (diluted 1:10,000, Proteintech, Rosemont, IL, 66379-1-IG), and AQP-1 (diluted 1:4,000, Proteintech, Rosemont, IL, 20333-1-AP), were added, and the samples were incubated at 4 °C overnight. After several washes, the samples were incubated with goat anti-rabbit or mouse secondary antibodies (1:20,000, Biosharp, Beijing, China, BL003A/BL001A) for 2 h at room temperature. The band intensity was analyzed using ImageJ software (Bethesda, MD) and normalized to the band intensity of GAPDH. The protein ladder was purchased from Biotides (Somerset, NJ) (Catalog No. WB19025).

### 2.9. Statistical analysis

GraphPad Prism 9.0 (La Jolla, CA) was used for data analysis. The quantitative data are presented as the means ± SD. Spearman’s correlation analysis was used to assess the correlation between tRF-1-ArgTCG-1-1 and clinical parameters. One-way analysis of variance (ANOVA) was used for comparisons of multiple groups. *p* < 0.05 was considered statistically significant.

### 2.10. Ethics approval

All human sample research was approved by the Medical Ethics Committee of Wannan Medical College (Approval Number: 2022LS No. 77) and performed according to the Declaration of Helsinki. All participants signed an informed consent form.

## 3. Results

### 3.1. Magnetic bead sorting of renal tubular epithelial cells

**Figure 1.**
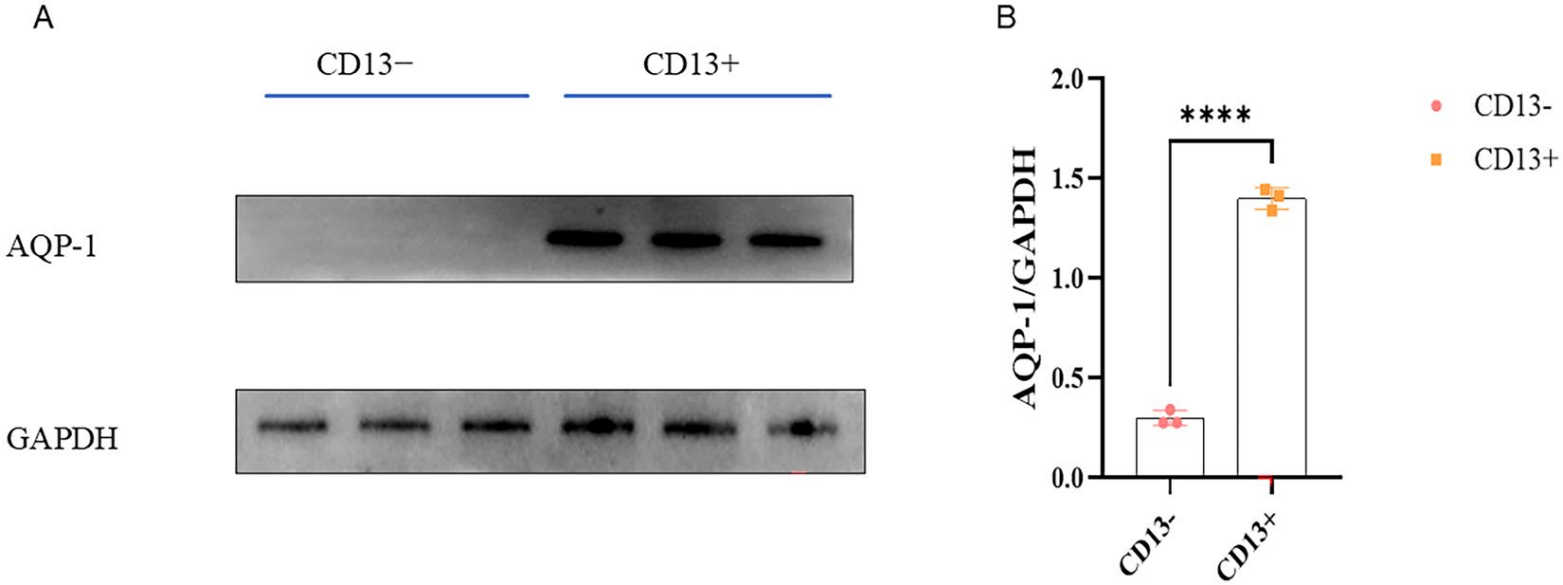
Magnetic bead sorting of renal tubular epithelial cells. (A) Western blot showed the presence of AQP-1 expression in the magnetic bead sorted CD13^+^ cells. (B) Quantitative analysis of AQP-1 (*n* = 3) (****p < 0.0001).

Magnetic bead sorting was applied to separate renal tubular epithelial cells from human urine sediment, and Western blot analysis was utilized to validate the sorting results. The Western blot results demonstrated that CD13^+^ cells expressed AQP-1 (Figure 1(A,B)).

### 3.2. Profiling and biological analysis of the human tRF microarray

**Figure 2.**
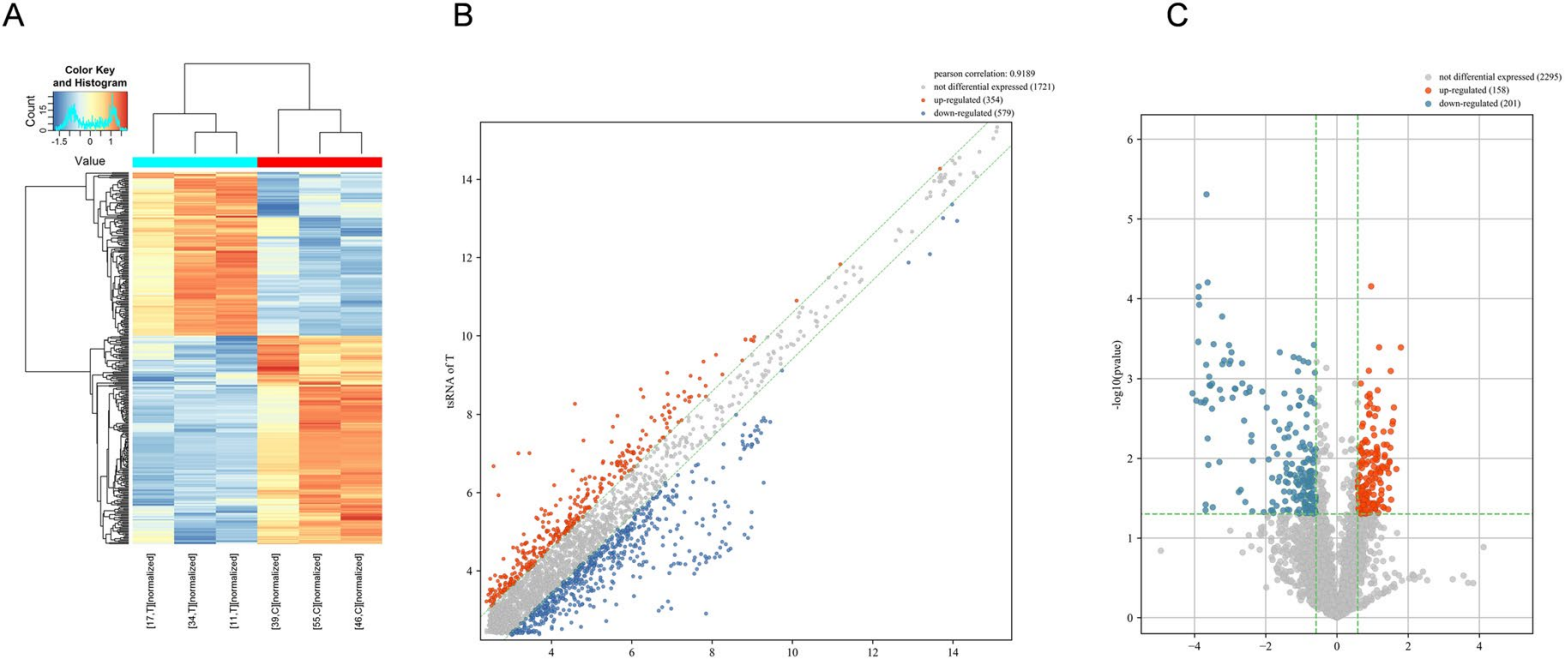
Differential tRFs expression profiles. (A) Clustered heat map, (B) scatter plot of the differentially expressed tRFs in three pairs of fibrosis and non-fibrosis renal tubular epithelial cells. (C) Volcano plots of the differentially expressed tRFs in three pairs of fibrosis and non-fibrosis renal tubular cells.

Clinical data, including clinical and pathological data, were collected from three patients without RF and three patients with RF in CKD. The data are shown in Supplementary Table S1. Overall, 359 tRFs were differentially expressed (FC >2 vs. control, *p* < 0.05), of which 158 were upregulated and 201 were downregulated. These genes were displayed by a hierarchical clustering heatmap, scatter plot, and volcano plot (Figure 2(A–C)).

**Figure 3.**
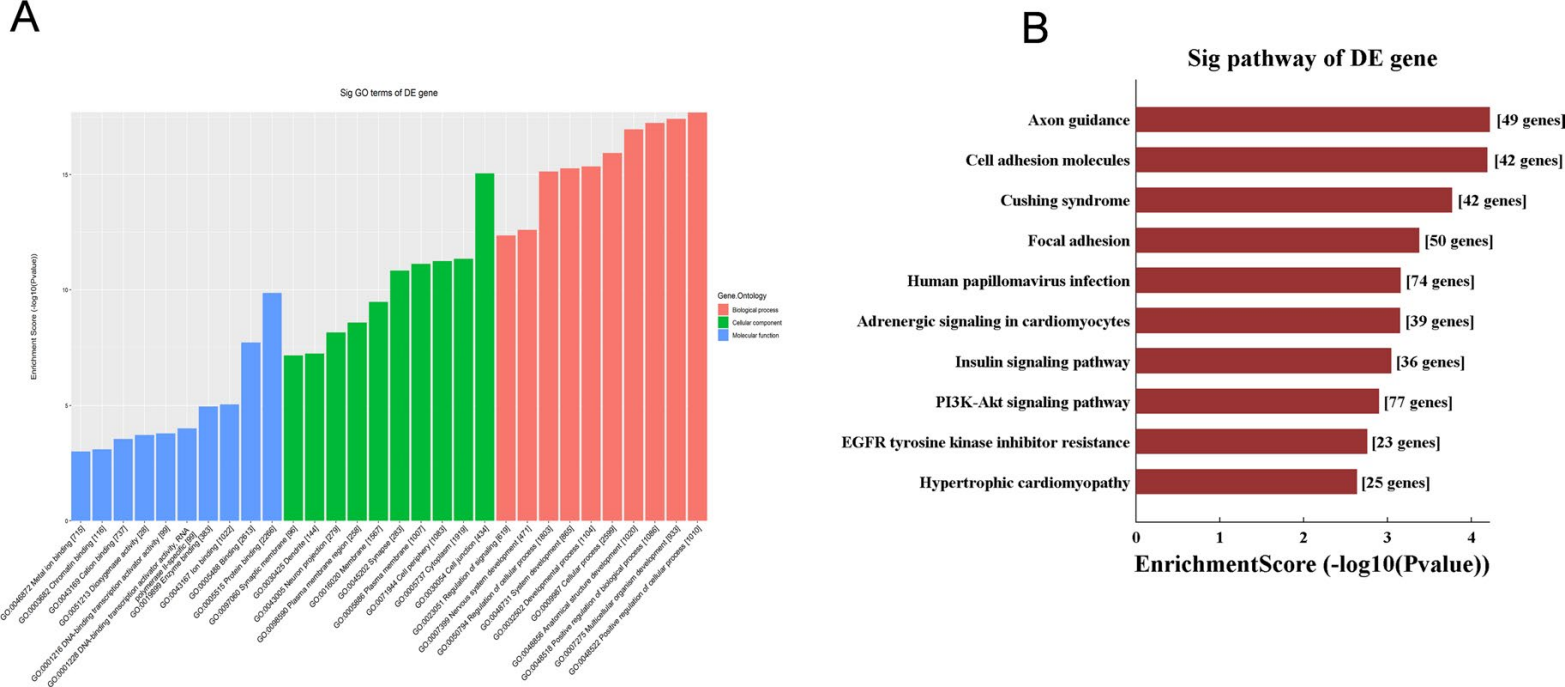
Biological analysis of predicted genes of differential expressed tRFs. (A) GO analysis of differential expressed genes. (B) KEGG pathway enrichment analysis of differential expressed.

The differentially expressed tRFs were forwarded for further bioinformatic analysis. The results of the Gene Ontology (GO) and Kyoto Encyclopedia of Genes and Genomes (KEGG) analyses are shown in Figure 3(A,B). Considering the limitation of small sample size in this exploratory screening cohort, which may increase the risk of false-positive results, we focused on the significantly upregulated tRF‑1‑ArgTCG‑1‑1 for further RT‑qPCR validation in an expanded independent cohort.

### 3.3. Clinical and pathological data on urinary renal tubular epithelial cells in patients with CKD

Sixty-six patients with CKD were categorized into three groups according to the extent of RF: none (fibrosis area 0–25%); mild to moderate (26–50%); and severe: (≥50%).

In the non-fibrosis group, the main types were IgA nephropathy (*n* = 3), membranous nephropathy (MN, *n* = 3), minimal change disease of the glomerulus (MCD, *n* = 2), purpura nephritis (*n* = 1), and lupus nephritis (*n* = 1).

In the mild-to-moderate fibrosis group, IgA nephropathy was predominant (*n* = 13), followed by MCD (*n* = 4), MN (*n* = 4), diabetic nephropathy (*n* = 3), hypertensive nephrosclerosis (*n* = 2), and a few rare entities including lupus nephritis, thin basement membrane nephropathy, acute interstitial nephritis, and renal amyloidosis (*n* = 1).

In the severe fibrosis group, advanced IgA nephropathy was the most common (*n* = 6), accompanied by diabetic nephropathy (*n* = 4), MN (*n* = 1), crescentic glomerulonephritis (*n* = 1), obesity-related glomerulomegaly (*n* = 1), and other proliferative/sclerosing or tubulointerstitial lesions (*n* = 2).

**Figure 4.**
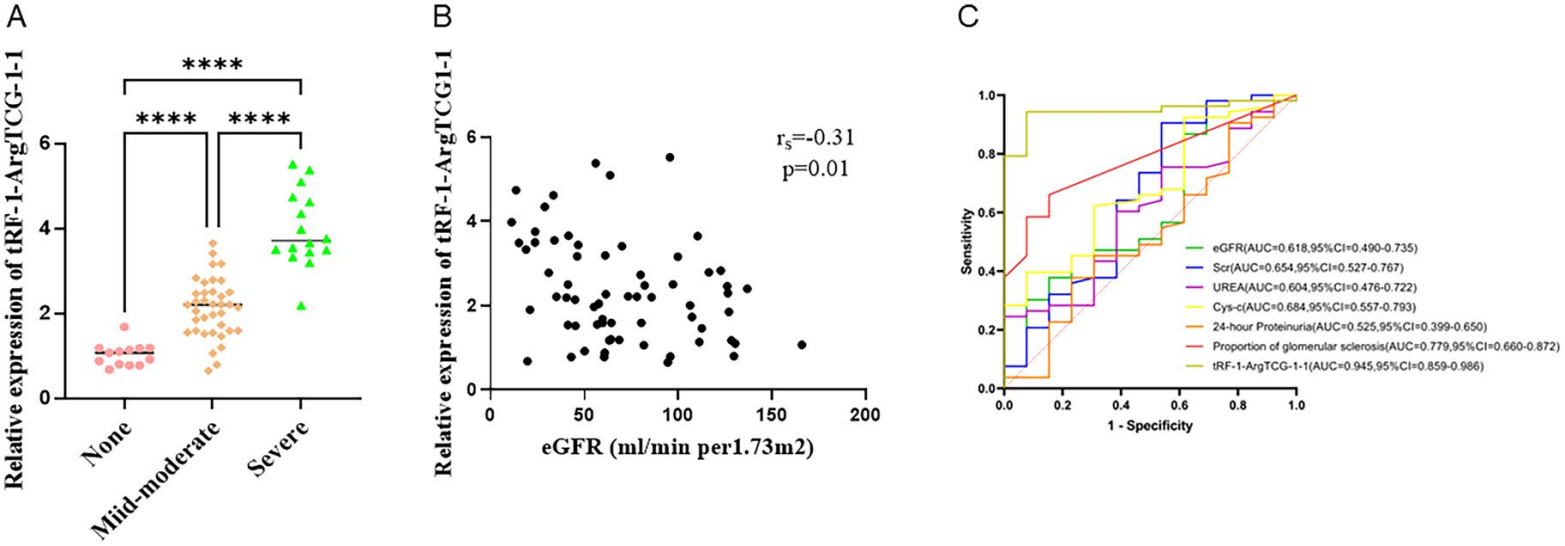
Diagnostic validation of tRF-1-ArgTCG-1-1 in renal fibrosis. (A) The expression of human urinary renal tubular epithelial cell-derived tRF-1-ArgTCG-1-1. (B) Correlation analysis between the expression of tRF-1-ArgTCG-1-1 and eGFR. (C) ROC analysis of tRF-1-ArgTCG-1-1 (****p < 0.0001).

The results revealed that the eGFR, Scr, cystatin C, and blood phosphorus levels, and glomerulosclerosis rate were significantly different (*p* < 0.05; Table 1). RT‐qPCR analysis revealed that the expression of human urinary renal tubular epithelial cell-derived tRF-1-ArgTCG-1-1 was highest in the severe fibrosis group Figure 4(A)). Correlation analysis revealed that tRF-1-ArgTCG-1-1 expression levels were negatively correlated with eGFRs (*r*_s_ = −0.315, *p* = 0.01) (Figure 4(B)). In addition, we constructed a multivariate logistic regression model.

**Table 1.** Clinical parameter of all patients involved.

	None (*n* = 13)	Mild (*n* = 37)	Moderate-severe (*n* = 16)	*p*
Sex (male/female)	5/8	24/13	14/2*^,#^	0.022
Age (years)	41.62 ± 18.33	48.00 ± 15.36	49.37 ± 15.78	0.369
Use of ACEI/ARB	4/9	17/20	5/11	0.469
Use of SGLT2	1/12	10/27	4/12	0.348
Use of ARNI	0/13	4/33	2/14	0.437
SBP (mmHg)	128.00 (101–153)	135.50 (82–169)	146.50 (128–214)*^,#^	0.002
DBP (mmHg)	77.92 ± 10.92	85.68 ± 12.52*	92.44 ± 13.42*^,#^	0.011
eGFR (mL/min per 1.73 m^2^)	63.20 (19.58–165.87)	79.84 (20.90–136.70)	33.64 (11.15–95.45)*^,#^	<0.001
Scr (μmol/L)	72.7 (37.7–342.1)	84.0 (47.4–212.6)*	193.3 (62.1–648.2)*^,#^	<0.001
UREA (mmol/L)	5.61 (2.51–9.53)	6.27 (3.19–22.91)	10.28 (4.02–24.88)*^,#^	0.006
UA (μmol/L)	354.88 ± 99.12	384.70 ± 90.83	427.44 ± 80.90	0.097
Cys-C (mg/L)	1.18 (0.55–1.77)	1.21 (0.61–2.61)	1.99 (0.93–3.83)*^,#^	<0.001
24-h proteinuria (g/day)	1.91 (0.11–10.82)	1.46 (0.05–20.99)	2.83 (0.34–9.64)	0.183
ALB (g/L)	31.65 ± 8.79	36.78 ± 8.53*	31.15 ± 7.34^#^	0.038
Glu (mmol/L)	4.80 (3.87–5.57)	4.97 (3.85–8.02)	4.87 (3.47–8.34)	0.597
Cho (mmol/L)	4.85 (3.30–10.15)	4.82 (3.22–10.71)	4.26 (3.22–8.24)	0.368
TG (mmol/L)	1.64 (0.92–2.40)	1.74 (0.59–4.33)	1.37 (0.57–2.96)	0.200
P (mmol/L)	1.28 (1.05–1.75)	1.07 (0.55–1.70)*	1.27 (0.95–2.11)^#^	<0.001
Hgb (g/L)	129.00 ± 24.34	128.81 ± 29.37	111.50 ± 22.24	0.088
Glomerular sclerosis ratio	0.02 (0.00–0.20)	0.10 (0.00–0.58)*	0.53 (0.00–0.88)*^,#^	<0.001
tRF-1-ArgTCG-1-1	1.03 ± 0.27	2.12 ± 0.69*	3.99 ± 0.90*^,#^	<0.001

UA: uric acid; Cyc-s: cystatin c; ALB: albumin; Glu: glucose; Cho: cholesterol; TG: triglycerides; P: phosphorus; Hgb: hemoglobin; SBP: systolic blood pressure; DBP: diastolic blood pressure; eGFR: estimated glomerular filtration rate; Scr: serum creatinine; UREA: blood urea nitrogen; ACEI: angiotensin-converting enzyme inhibitor; ARB: angiotensin II receptor blocker; SGLT2: sodium-glucose cotransporter 2; ARNI: angiotensin receptor-neprilysin inhibitor.

* *p* < 0.05 versus the none group (no fibrosis).

^#^ *p* < 0.05 versus the mild group (mild fibrosis).

Regression analysis revealed that after adjusting for sex as a confounding factor, only the expression of tRF-1-ArgTCG-1-1 (OR = 154.30; 95%CI: 3.95–6,021.45; *p* = 0.007) was significantly associated with the severity of RF (Table 2). Each unit increase in the expression of tRF-1-ArgTCG-1-1 was associated with a 154-fold higher risk of RF. In contrast, sex, Scr, urea nitrogen, cystatin C levels, eGFR, and 24-h proteinuria were not statistically significant in the multivariate logistic regression analysis (*p* > 0.05). ROC analysis revealed superior diagnostic accuracy for tRF-1-ArgTCG-1-1 (AUC = 0.945, 95%CI: 0.859–0.986; *p* < 0.0001) compared with the glomerulosclerosis proportion (AUC = 0.779), cystatin C level (AUC = 0.684), and other clinical parameters. A relative expression level of urinary tRF-1-ArgTCG-1-1 at 1.197 could distinguish patients with fibrosis, with a sensitivity of 94.3% and a specificity of 92.3% (Figure 4(C)).

**Table 2.** Multivariate logistic regression analysis.

	OR	95%CI	*p* Value
Sex	7.33	0.53–100.99	0.137
tRF-1-ArgTCG-1-1	154.30	3.95–6,021.45	0.007
eGFR (ml/min per 1.73 m²)	1.01	0.93–1.10	0.779
Scr (mmol/L)	0.99	0.95–1.02	0.436
UREA (mmol/L)	1.32	0.59–2.97	0.505
Cys-c (mg/L)	6.26	0.00–64,601.34	0.697
24 h proteinuria (g/24 h)	0.68	0.41–1.12	0.131

Scr: serum creatinine; eGFR: estimated glomerular filtration rate; Cyc-s: cystatin c; UREA, blood urea nitrogen (BUN).

Female was used as the reference category in the logistic regression analysis.

Relative expression levels of tRF-1-ArgTCG-1-1 were calculated using the 2^-ΔΔCt^ method after normalization to the internal control.

### 3.4. tRF-1-ArgTCG-1-1 silencing attenuates the TGF‐β1‐induced expression of collagen I and α‐SMA in HK‐2 cells

**Figure 5.**
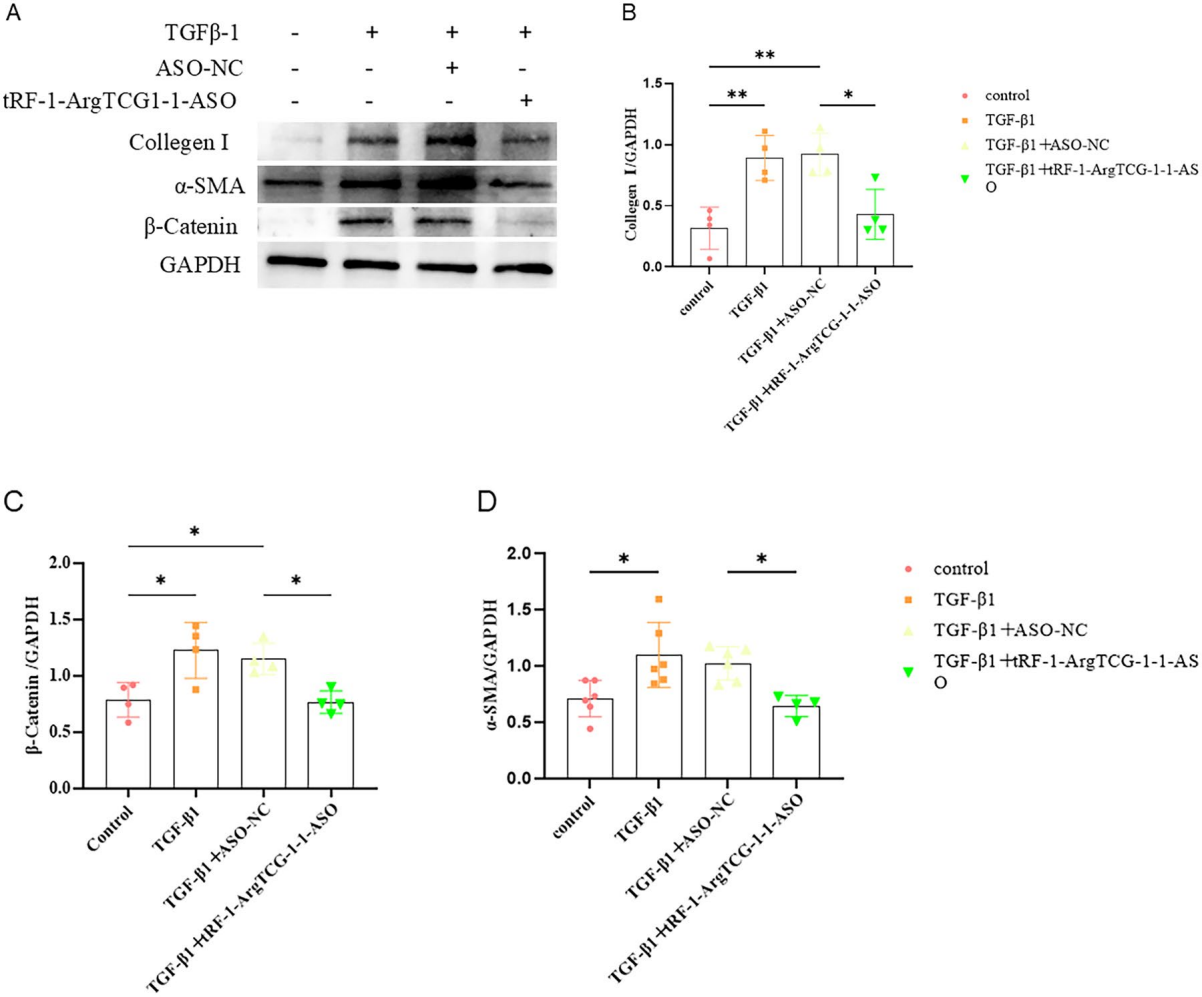
tRF-1-ArgTCG-1-1 silencing attenuates expression of collagen I, α-SMA, and β-catenin in TGF-β1 treated HK-2 cells. (A) Western blot showed that protein expression of collagen I, α-SMA, and β-catenin was inhibited by silencing tRF-1-ArgTCG-1-1 after transfection of siRNA in TGF­β1 treated HK2 cells. (B) Quantitative protein blotting plots of collagen I (*n* = 4). (C) Quantitative analysis of β-catenin (*n* = 4). (D) Quantitative analysis of α­SMA (*n* = 6) (*p < 0.05; **p < 0.01).

To investigate the impact of tRF-1-ArgTCG-1-1 silencing on fibrogenesis, HK‐2 cells were transfected with tRF-1-ArgTCG-1-1 ASO. Western blot (Figure 5(A–D)) and immunofluorescence (Figure 6(A–C)) results revealed a significant reduction in the expression of the fibrosis‐related marker proteins α‐SMA and collagen I after tRF-1-ArgTCG-1-1 was silenced compared with those in the TGF‐β1 without tRF-1-ArgTCG-1-1 ASO-treated group.

Figure 6.tRF-1-ArgTCG-1-1 silencing attenuates expression of collagen I, α-SMA and β-catenin in TGF-β1 treated HK-2 cells. (A) Decreased α-SMA fluorescence was detected by immunofluorescence confocal microscopy in HK2 cells with tRF-1-ArgTCG-1-1 ASO transfected (bar = 50 μm). (B) Decreased β-Catenin fluorescence was detected by immunofluorescence confocal microscopy in HK2 with cells with tRF-1-ArgTCG-1-1 ASO transfected. (C) Decreased collagen I fluorescence was detected by immunofluorescence confocal microscopy in HK2 cells with tRF-1-ArgTCG-1-1 ASO transfected (bar = 50 μm).

**Figure:**
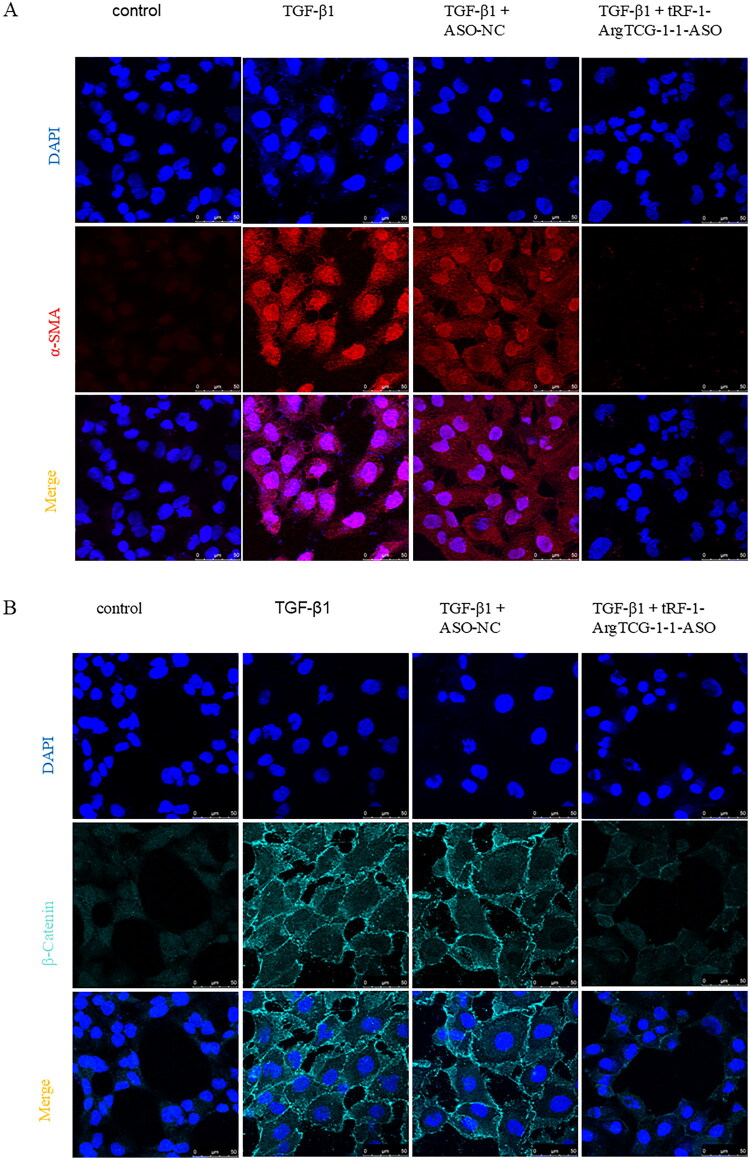

**Figure:**
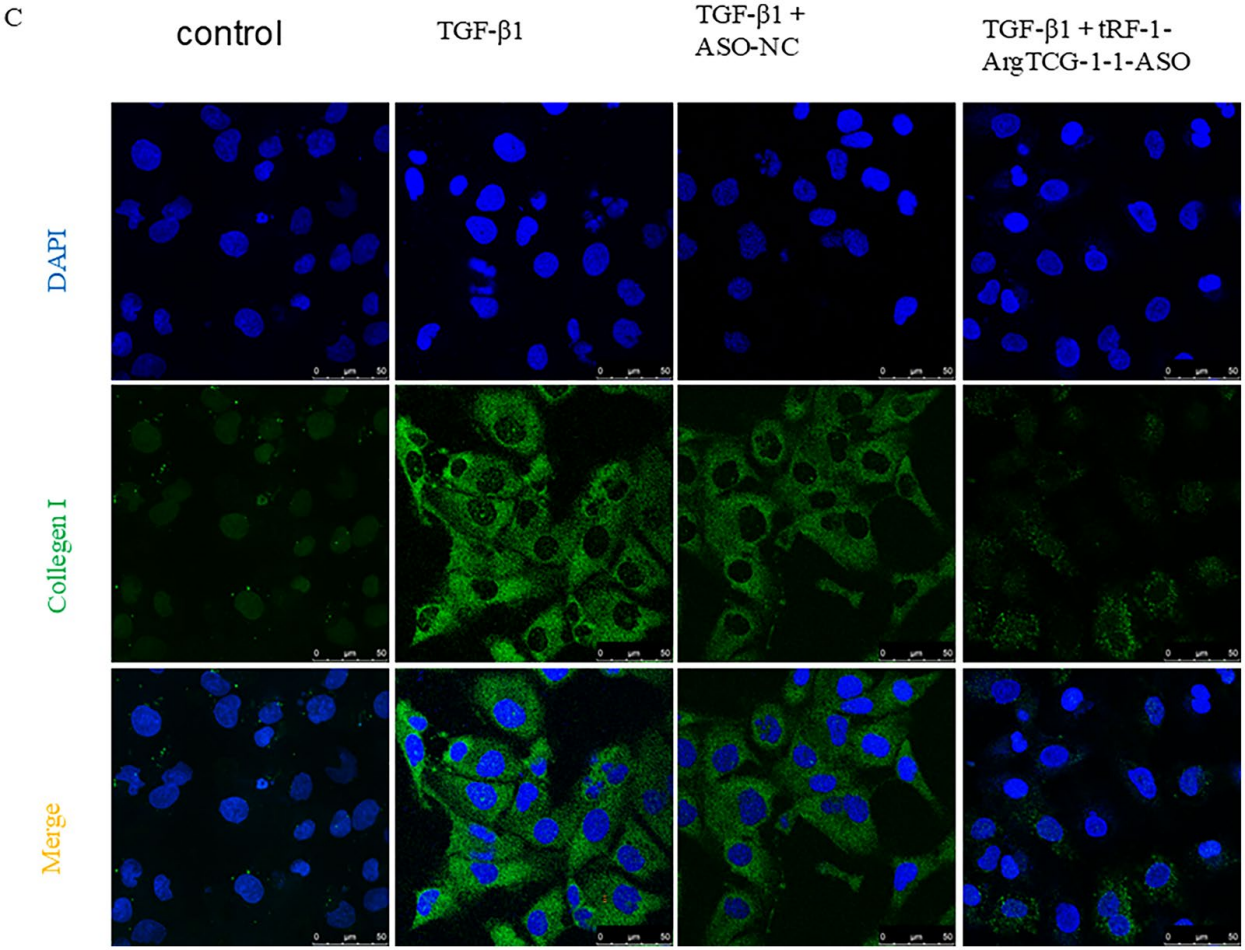

### 3.5. Identification and validation of tRF-interacting proteins

**Figure 7.**
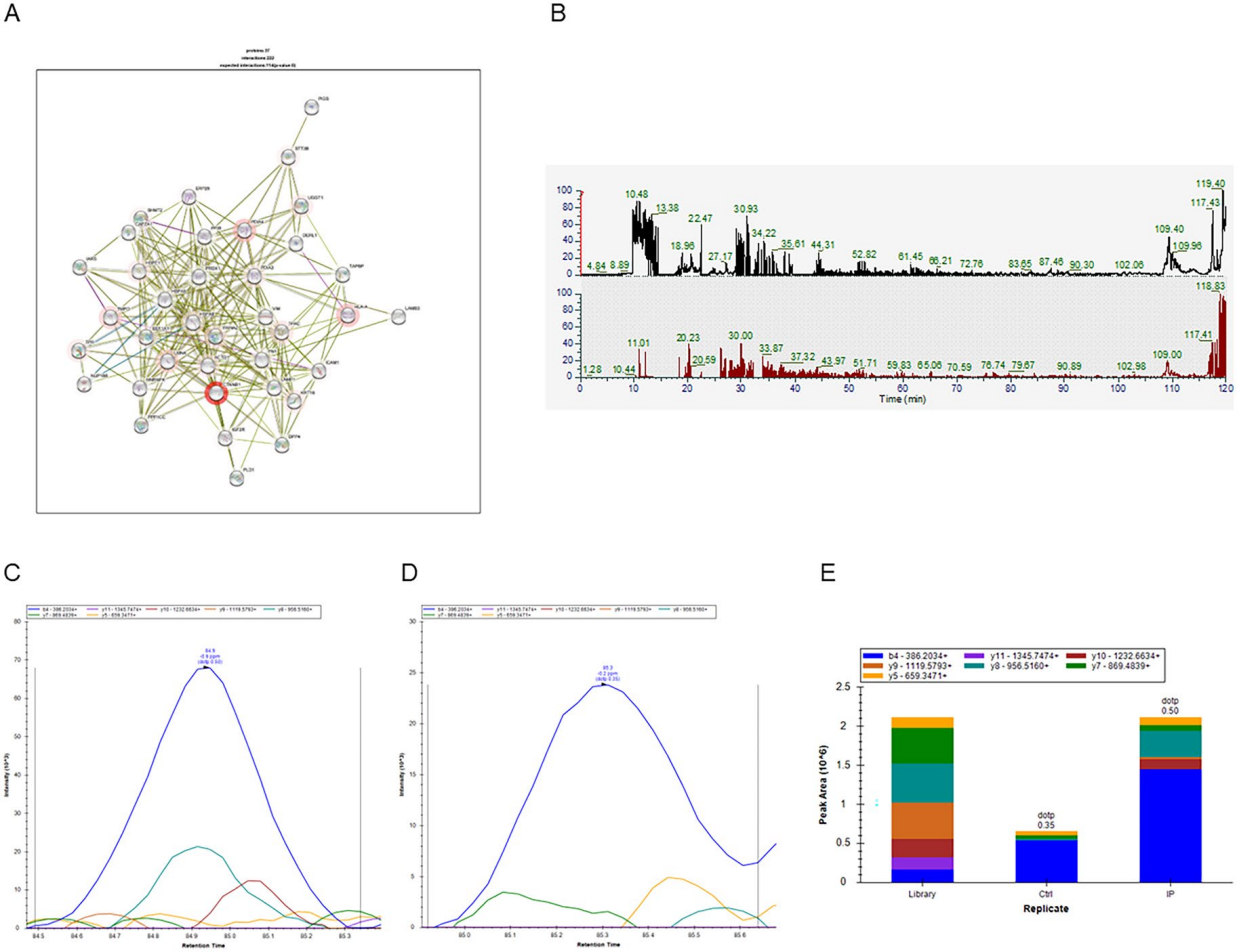
Plot of protein profiling experiments. (A) Search Tool for the Retrieval of Interacting Genes/Proteins Database (STRING DB) protein interaction analysis. Protein interaction analysis of T-specific binding proteins using Stringdb revealed significantly enriched interactions between 37 molecules. (B) Mass spectrometry data visualization plot. The mass spectrometry results showed the presence of mass peaks. (C) Peptide peak times and abundance values in control samples: the above figure shows the qualitative information of the polypeptide KLFHEVVQAFRA, the peptide abundance is the area that the fold line and the horizontal axis form. (D) Peptide peak times and abundance values in IP samples: the above figure shows the qualitative information of the polypeptide KLFHEVVQAFRA, the peptide abundance is the area that the fold line and the horizontal axis form. (E) Differential expression of polypeptide abundance levels: quantitative comparison bar chart of polypeptide KLFHEVVQAFRA among sample groups, column height indicates the level of peptide abundance.

Hydroxylamine-mediated protein precipitation mass spectrometry (Hypro-MS) was performed to identify the tRF-1-ArgTCG-1-1-interacting proteins. The results revealed that β-catenin was a target protein of tRF-1-ArgTCG-1-1 (Figure 7(A)). The mass spectrometry results revealed that the liquid phase and mass spectrometry systems were operating well during the identification process, with mass spectral peaks present in the IP and control samples (Figure 7(B)). Furthermore, to validate the Hypro-MS data, parallel reaction monitoring (PRM) analysis was performed. The results revealed that β-catenin interacted with tRF-1-ArgTCG-1-1 (Figure 7(C–E)). Western blot analysis also revealed that the expression of β-catenin increased in the TGF-β1-treated HK-2 cells. Silencing of tRF-1-ArgTCG-1-1 with an ASO significantly decreased β-catenin expression (Figure 5).

### 3.6. Expression of renal fibrosis protein markers after tRF-1-ArgTCG1-1 and β-catenin silencing

To further validate the involvement of tRF-1-ArgTCG1-1 in the regulation of RF through the β-catenin pathway, β-catenin siRNA sequences were constructed *in vitro*. The most effective siRNA sequence was selected (Figure S1). Treatment with tRF-1-ArgTCG1-1-ASO significantly decreased the expression of collagen I, β-catenin, and α-SMA. Administration of β-catenin–siRNA not only inhibited the expression of the β-catenin protein but also inhibited the expression of collagen I and α­SMA (Figure 8(A–D)). Immunofluorescence revealed that after treatment with tRF-1-ArgTCG1-1-ASO and β-catenin–siRNA, the expression of α-SMA, β-catenin, and collagen I significantly decreased in HK-2 cells (Figure 8(E–G)).

Figure 8.The expression of renal fibrosis protein markers after tRF-1-ArgTCG-1-1 and β-catenin silencing. (A) Western blot demonstrated the inhibition of protein expression of collagen I, α-SMA, and β-catenin in tRF-1-ArgTCG-1-1 ASO and siRNA-β-catenin transfected HK-2 cells under the treatment of TGF-β1. (B) Quantitative analysis of α­SMA. (C) Quantitative analysis of β-catenin. (D) Quantitative analysis of collagen I. (E) Immunofluorescence images showed down-regulation of α­SMA after transfection of tRF-1-ArgTCG-1-1 ASO and siRNA-β-catenin in TGF­β1 treated HK2 cells (bar = 50 μm). (F) Immunofluorescence images showed down-regulation of β-catenin after transfection of tRF-1-ArgTCG-1-1 ASO and siRNA-β-catenin TGF­β1 treated HK2 cells (bar = 50 μm). (G) Immunofluorescence images showed down-regulation of collagen I after transfection of tRF-1-ArgTCG-1-1 ASO and siRNA-β-catenin in TGF-β1 treated HK2 cells (bar = 50 μm) (*p < 0.05; **p < 0.01; ***p < 0.001; ****p < 0.0001).

**Figure:**
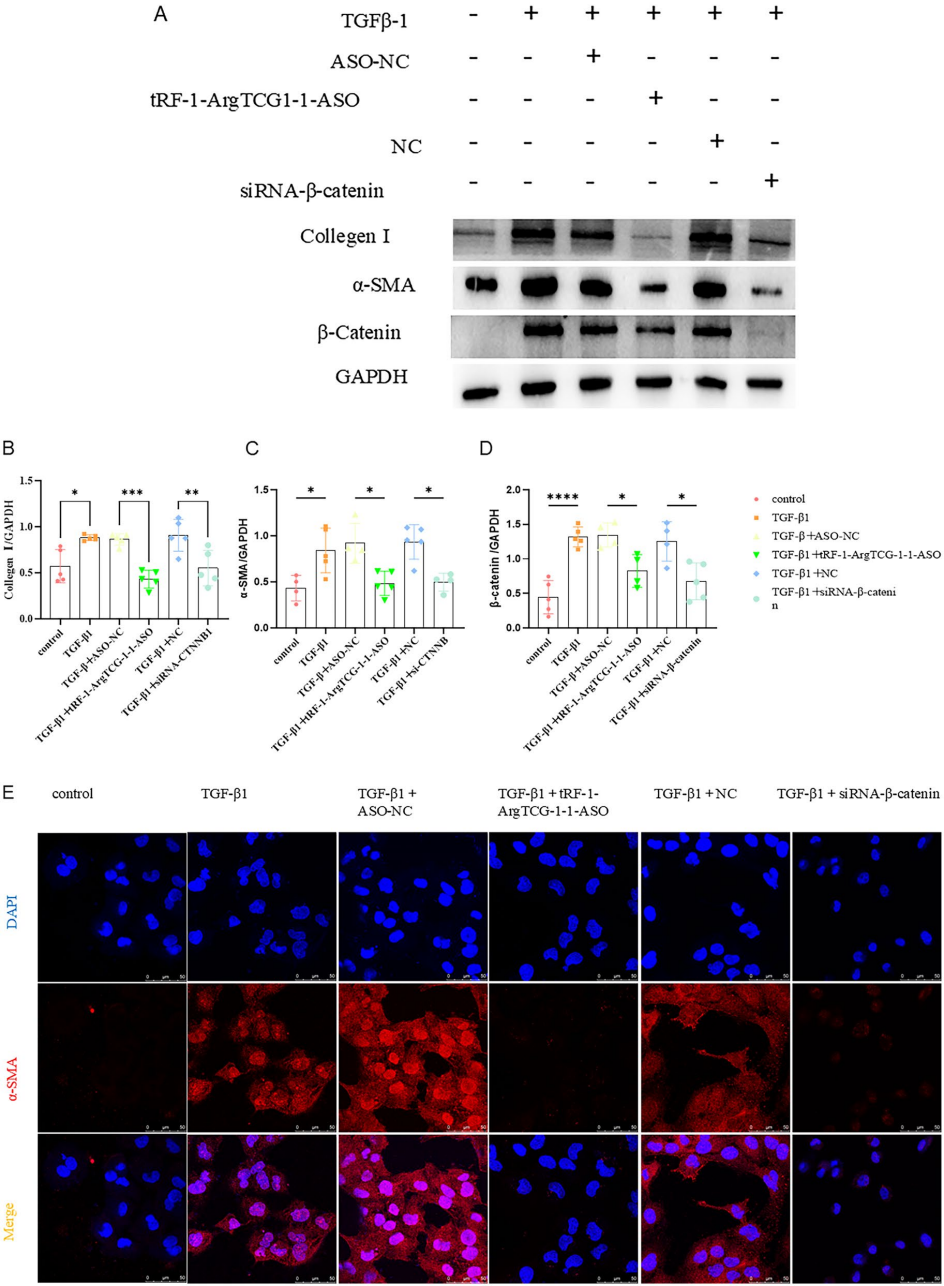

**Figure:**
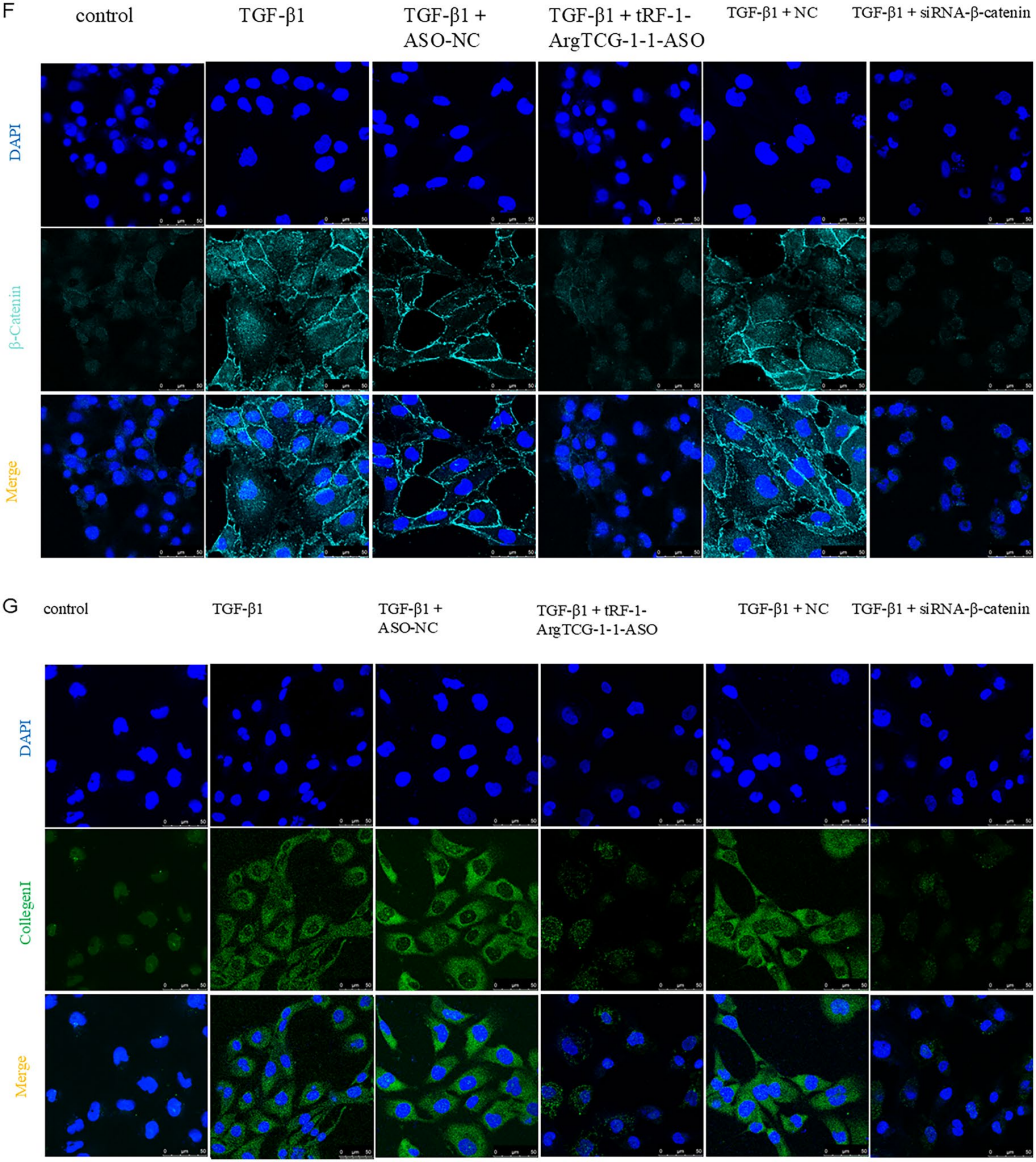

## 4. Discussion

In this study, we first revealed that the expression of urinary renal tubular epithelial cell-derived tRF-1-ArgTCG-1-1 was upregulated in patients with RF and further demonstrated that tRF-1-ArgTCG-1-1 promoted RF via interaction with β-catenin. tRF-1-ArgTCG-1-1/β-catenin is a potential therapeutic target for CKD and RF.

Renal fibrosis is a common cause of end-stage renal diseases [4]. Early diagnosis is crucial for slowing the progression of CKD [23]. Aquaporin water channels are a family of membrane proteins that facilitate water movement across biological membranes. Previous studies have reported that AQP1 (Aquaporin 1) can be detected in tubular epithelial cells shed into urine, it has been widely utilized in research on tubular injury and related diseases [24].

tRNA-derived fragments are noncoding short RNAs that play significant biological roles and were once thought to be random byproducts of tRNA breakdown [25,26]. Over the past decades, extensive research has been devoted to uncovering the roles of ncRNAs in RF, such as miR-21 [27], MALAT1 [28], have been shown to regulate fibrogenic pathways. Recently, multiple studies have shown that tRFs play key roles in cell proliferation [29], differentiation [19], apoptosis, and survival [30]. An increasing number of studies have shown that tRFs may participate in the progression of kidney diseases. Mishima et al. introduced novel insights into the constitutive alterations in tRFs, suggesting that tRFs could serve as an intervention marker for CKD [31]. Additionally, earlier studies suggested that urinary exosomal tRFs might be new biomarkers for CKD diagnosis [18]. Li et al. emphasized the dysregulation of tRF-Val-TAC-004 in the IRI-AKI paradigm and demonstrated how tRF-Val protects against IRI-AKI by blocking Apaf1-mediated apoptosis [20,21]. In this study, we revealed the expression profiles of urinary renal tubular epithelial cells in patients with RF and further revealed that the expression of tRF-1-ArgTCG-1-1 is strongly related to renal function and the risk of RF. Moreover, the role of tRF-1-ArgTCG-1-1 was further validated in TGF-β1-treated HK-2 cells. Silencing of tRF-1-ArgTCG-1 significantly attenuated the expression of RF marker proteins. Accordingly, renal tubular epithelial cell-derived tRF-1-ArgTCG-1-1 may play a vital role in RF.

tRF has been shown to regulate the progression of several diseases via interactions with different proteins. Green et al. reported that tRF-GlyTCC interacted with runt-related transcription factor 2 (RUNX2) and Y-box-binding protein 1 (YBX1) in bone cancer. An earlier study also indicated that tRF-3019a binds to Ago2, which regulates the expression of FBXO47 in gastric cancer [32]. In breast cancer, 5′ tiRNAVal binds to the 3′UTR of FZD3 and suppresses FZD3-mediated Wnt/β-catenin signaling [33]. In our study, we also aimed to identify the protein that interacts with tRF-1-ArgTCG-1-1. According to the data from the Hypro-MS and PRM assays, β-catenin is the protein that interacts with tRF-1-ArgTCG-1-1. The Wnt/β-catenin pathway is a widely researched signaling pathway that plays a role in renal growth and repair [34]. Wnt/β-catenin signaling is not activated in healthy individuals. However, in different forms of CKD, Wnt/β-catenin signaling is reactivated [35]. Chen et al. revealed that β-catenin prevents LKB1 from being SUMOylated, which disrupts fatty acid oxidation in renal tubular cells and leads to RF [36].

The primary mediator of Wnt signaling, β-catenin, is thought to be primarily regulated at the posttranslational level by phosphorylation and ubiquitination-mediated degradation [37]. In our study, we discovered that β-catenin has a profibrotic role in RF, indicating its involvement in the progression of RF. More interestingly, the expression of β-catenin was regulated by tRF-1-ArgTCG-1-1. Silencing tRF-1-ArgTCG-1-1 inhibited expression of β-catenin in TGF-β1-treated HK-2 cells. These discoveries provide fresh perspectives on the small RNA-mediated molecular processes involved in RF and may provide potential therapeutic targets in CKD.

Our study has several limitations. First, the validation of human small RNA microarray results was based on a relatively small sample size and lacked sufficient follow-up analyses. Moreover, sex imbalance among different fibrosis groups represents a potential confounder. Thus, larger and more balanced cohorts are needed in future research to validate the present findings. Second, the specific molecular mechanism by which tRF-1-ArgTCG-1-1 regulates β-catenin expression was not elucidated. Whether this regulation occurs at the transcriptional, post-transcriptional, or protein stability level requires further investigation. Third, a limitation of this study is that rescue assays were not included to determine whether restoration of β-catenin could reverse the antifibrotic effect induced by tRF-1-ArgTCG-1-1 silencing. Future studies incorporating β-catenin rescue experiments will be important to more conclusively define the tRF-1-ArgTCG-1-1/β-catenin regulatory axis. Furthermore, to date, no reasonable homologue of tRF-1-ArgTCG-1-1 in mice has been found in the existing tsRNA database, which hinders *in vivo* experimental validation. Future studies will employ a kidney organ-on-a-chip platform to further evaluate the functional role of tRF-1-ArgTCG-1-1 in RF.

## 5. Conclusions

This study revealed that tRF-1-ArgTCG1-1 plays a critical role in the progression of RF by directly interacting with β-catenin. These results provide evidence that active tRFs can function as regulators of RF and act as therapeutic targets for CKD and RF.

## Supplementary Material

Supplemental Material

Supplemental Material

## Data Availability

All data produced or analyzed during this study are incorporated in this article or displayed in supplementary material. The raw data of experiments used to support the findings of this study are available from the corresponding author upon request.
