# Supplementary material for: tRF-1-ArgTCG-1-1 promotes renal fibrosis by regulating β-catenin
Source: Ren Fail. 2026 Jun 1;48(1):2670055. doi: 10.1080/0886022X.2026.2670055 (PMC13228170; doi:10.1080/0886022X.2026.2670055)
Supplement: Supplemental Material [file IRNF_A_2670055_SM7553.docx]

Table S1. Clinical information on 3 patients without fibrosis and 3 patients with fibrosis

|  | None fibrosis(n＝3） | | |  | fibrosis(n＝3） | | | P |
| --- | --- | --- | --- | --- | --- | --- | --- | --- |
|  | Patient1 | Patient2 | Patient3 |  | Patient1 | Patient2 | Patient3 |  |
| Age | 73 | 58 | 54 |  | 57 | 42 | 43 | 0.130 |
| 24h Proteinuria  (g/day) | 7.27 | 2.39 | 3.19 |  | 2.87 | 1.71 | 1.62 | 0.229 |
| Scr (mmol/L) | 561.6 | 331 | 104.8 |  | 37.7 | 44.4 | 161.3 | 0.142 |
| eGFR (ml/min per1.73m2) | 15 | 23.6 | 47 |  | 68.5 | 108.9 | 50.1 | 0.075 |
| UREA (mmol/L) | 17.42 | 10.43 | 8.53 |  | 4.78 | 5.45 | 9.53 | 0.147 |
| UA (μmol/L) | 363.8 | 209.9 | 303.9 |  | 247.6 | 438.1 | 466.2 | 0.327 |
| MCHC（g/L） | 332 | 353 | 335 |  | 334 | 336 | 358 | 0.805 |
| Hb（g/L） | 76 | 103 | 136 |  | 113 | 100 | 122 | 0.737 |
| cyc-s(mg/L) | 3.32 | 2.47 | 1.41 |  | 1.11 | 0.74 | 1.51 | 0.098 |
| Glu(mmol/L) | 3.97 | 5.05 | 10.46 |  | 4.82 | 4.63 | 4.25 | 0.393 |

Scr: serum creatinine; GFR: estimated glomerular filtration rate; UA: uric acid; Cyc-s: cystatin c; Glu: glucose.
