# Supplementary figures and images for: tRF-1-ArgTCG-1-1 promotes renal fibrosis by regulating β-catenin
Source: Ren Fail. 2026 Jun 1;48(1):2670055. doi: 10.1080/0886022X.2026.2670055 (PMC13228170; doi:10.1080/0886022X.2026.2670055)

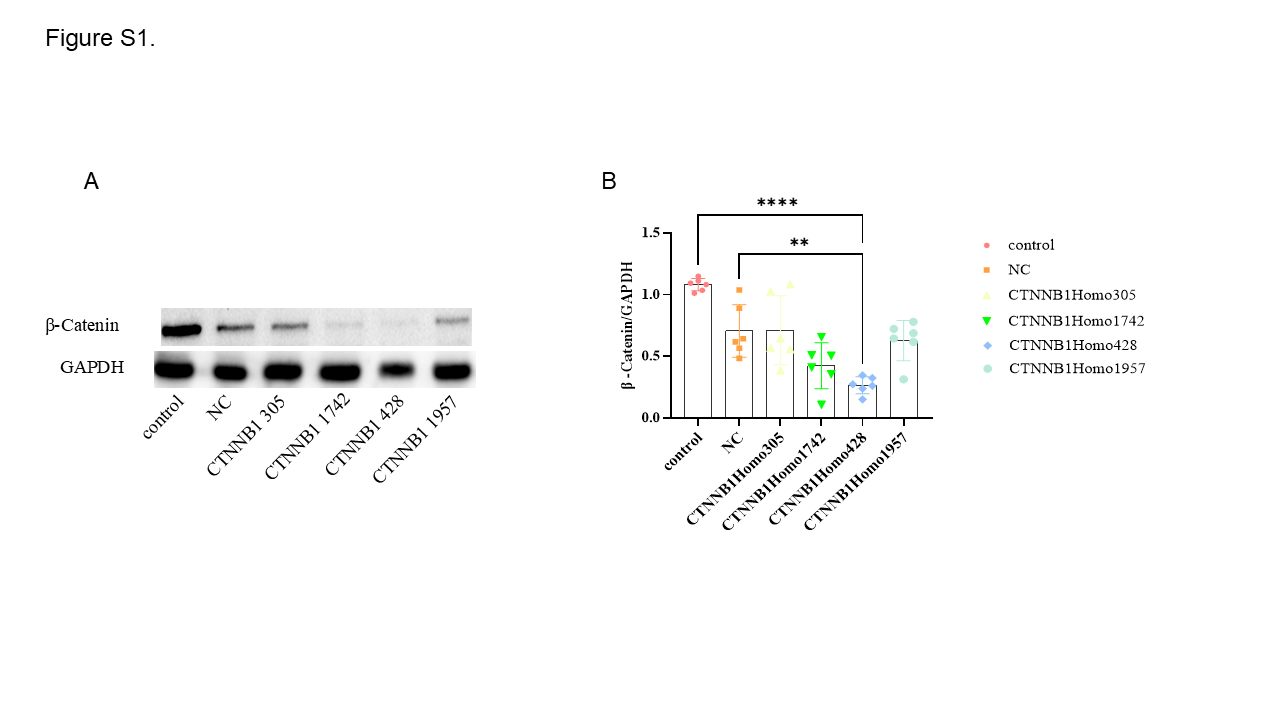

Supplement: Supplemental Material [file IRNF_A_2670055_SM7549.tif]
